# Characterization of Sustainable Robotic Materials and Finite Element Analysis of Soft Actuators Under Biodegradation

**DOI:** 10.3389/frobt.2021.760485

**Published:** 2021-11-24

**Authors:** Toshiaki Nagai, Ashitaka Kurita, Jun Shintake

**Affiliations:** Department of Mechanical and Intelligent Systems Engineering, School of Informatics and Engineering, The University of Electro-Communications, Chofu, Japan

**Keywords:** soft robotics, green robotics, sustainable, biodegradable, soft actuators, finite element analysis

## Abstract

Biodegradability is an important property for soft robots that makes them environmentally friendly. Many biodegradable materials have natural origins, and creating robots using these materials ensures sustainability. Hence, researchers have fabricated biodegradable soft actuators of various materials. During microbial degradation, the mechanical properties of biodegradable materials change; these cause changes in the behaviors of the actuators depending on the progression of degradation, where the outputs do not always remain the same against identical inputs. Therefore, to achieve appropriate operation with biodegradable soft actuators and robots, it is necessary to reflect the changes in the material properties in their design and control. However, there is a lack of insight on how biodegradable actuators change their actuation characteristics and how to identify them. In this study, we build and validate a framework that clarifies changes in the mechanical properties of biodegradable materials; further, it allows prediction of the actuation characteristics of degraded soft actuators through simulations incorporating the properties of the materials as functions of the degradation rates. As a biodegradable material, we use a mixture of gelatin and glycerol, which is fabricated in the form of a pneumatic soft actuator. The experimental results show that the actuation performance of the physical actuator reduces with the progression of biodegradation. The experimental data and simulations are in good agreement (*R* ^2^ value up to 0.997), thus illustrating the applicability of our framework for designing and controlling biodegradable soft actuators and robots.

## Introduction

Recently, there has been increasing research interest in the field of soft robotics, where robotic systems and elements are fabricated from compliant materials (Rus and Tolley, 2015; Polygerinos et al., 2017; Rich et al., 2018; Shintake et al., 2018). Owing to such inherent compliance and relatively simple structures, soft robots offer high mechanical robustness, safety to humans, and adaptability to the surrounding environment. One of the promising applications in soft robotics involves mobile robots that perform tasks during search and rescue operations in disaster areas and exploration of the natural environment (Tolley et al., 2014; Galloway et al., 2016; Katzschmann et al., 2018). In such tasks, deploying a large number of robots is an effective strategy for covering wide areas in limited amounts of time. However, many soft robots are designed to operate in the natural environment; hence, there is a possibility that they may become waste because of unexpected accidents, resulting in environmental degradation.

One method of addressing this issue is to endow soft robots with biodegradability, which enables them to naturally return to the soil in an environmentally friendly manner. In addition, many biodegradable materials are natural in origin, and creating soft robots using such materials would enhance sustainability. Given the promotion of sustainable development goals (SDGs) and international efforts thereof, the development of soft biodegradable materials and robotic systems is an important research avenue that is expected to contribute toward expanding the use of robots in the future. In this context, researchers have proposed strategies to create soft actuators and robots based on biodegradable materials (Je and Kim, 2004; Stroganov et al., 2014, 2015; Rossiter et al., 2016; Shintake et al., 2017; Zolfagharian et al., 2017, 2018; Baumgartner et al., 2020; Hughes and Rus, 2020). Some studies have shown that biodegradable soft actuators exhibit comparable performance with those based on conventional materials, such as silicone elastomers, and that biodegradable materials have excellent mechanical characteristics in terms of stretchability and robustness to repeated actuation cycles.

Biodegradable materials change their mechanical properties along with degradation by microorganisms (Dalev et al., 2001; Shogren et al., 2003; Abd El-Rehim et al., 2004; Martucci and Ruseckaite, 2009a; González et al., 2011; Nagai and Shintake, 2019). This indicates that the behaviors of actuators may differ based on the progression of degradation, where the outputs may not always remain consistent against identical inputs. Therefore, to appropriately operate biodegradable soft actuators and robots, it is necessary to reflect changes in their properties in the design and control processes. However, there is insufficient insight on how biodegradable actuators change their actuation characteristics and how these changes may be identified.

In this study, we attempt to address this problem by building and validating a framework to clarify the changes in the mechanical properties of biodegradable materials that allows prediction of the actuation characteristics of degraded soft actuators. In the proposed framework, a simulated environment of a soft biodegradable actuator is developed, wherein the mechanical properties of the material are implemented as functions of the degradation rate. We then characterize a mixture of gelatin and glycerol as a soft robotic biodegradable material and subsequently build its simulated environment as a design method for the actuator. Finally, we validate the simulation by comparisons with a physical actuator.

## Materials and Methods

**FIGURE 1:**
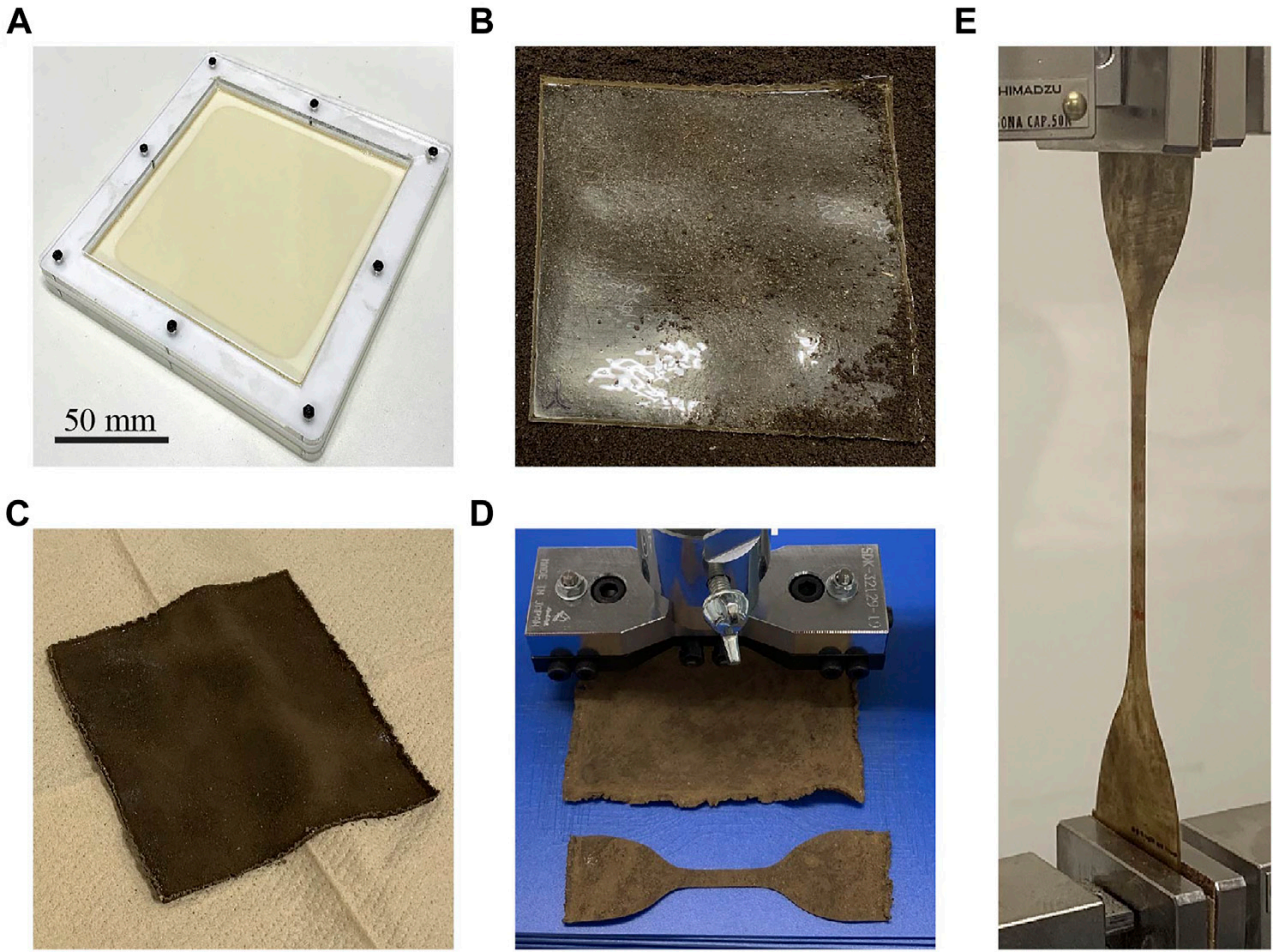
Characterization of gelatin–glycerol films. **(A)** Preparation of a film sample using a mold; the mixture of film material is already poured in the mold. **(B)** Film sample placed on the soil (just before commencement of biodegradation). **(C)** Degraded film sample removed from the soil. **(D)** Punching out the film sample. **(E)** Tensile testing of the specimen.

We chose a mixture of gelatin and glycerol as a soft robotic biodegradable material that could be formed as a film sample and degraded by soil. Another reason for selecting this material was that it could be degraded quickly, allowing for accelerated experiments in an extreme case. Therefore, it should be noted that degradation is expected to be relatively slow in practical situations. Additionally, the lifetime of a material can be greatly increased by coating it with environmentally friendly materials such as shellac, as reported in the work of Baumgartner et al. (2020), where a sample with the same composition as our material showed no dissolution after 48 h of immersion in water or 24 h in an acidic solution. During the biodegradation process, the mechanical properties of the sample were characterized through tensile tests. Further, the biodegradation rate was acquired, and its relationship with the mechanical properties was clarified. Figure 1 summarizes the process flow of the experiment.

The mechanical properties were obtained from the characterization as functions of the degradation rate and implemented using a finite element analysis software as the material constants to simulate the actuation behaviors of a soft actuator during biodegradation. We chose the pneumatic configuration as the type of actuation principle, where the actuator undergoes bending upon air pressurization. The simulation results were experimentally validated by characterizing a physical actuator with identical shape and dimensions as the simulated device. The physical actuator was made of the same material as that characterized in the process shown in Figure 1 and was subjected to biodegradation. Meanwhile the actuator was characterized via measurement of the actuation curvature as a function of the input pressure for different degradation rates.

### Preparation of Film Samples

Gelatin powder (17009-01, Kanto Chemical) and glycerol (17029-00, Kanto Chemical) were purchased from a supplier. The gelatin powder (Gel), glycerol (Gly), and distilled water (Wat) were mixed in a mass ratio of Gel:Gly:Wat = 2:1:8, placed in a beaker, and left to stand for 10 min to soften the gelatin powder. The solution was then stirred at 200 rpm at 80°C for 30 min to completely mix the ingredients. Subsequently, the mixture was poured into a set of molds that have internal dimensions of W 110 mm × L 110 mm (Figure 1A). Each mold was filled with 30 g of the solution. After removing the air bubbles by spraying ethanol, the samples were cured at 40°C for 2 h in an oven to form films with an average thickness of ∼0.5 mm. The films were then carefully removed from the molds and stored in a humidity chamber (WET-297-AHU, Tolihan) at room temperature (∼24°C) and 67% relative humidity (RH).

### Biodegradation of Film Samples

The soil used in this study was collected from our university campus and filtered using a 2 mm sieve. The measured moisture ratio of the soil was 63.4 ± 5.4% [*N* = 9, in accordance with JIS A1203, ISO 17892-1 (ISO 17892-1:2014. International Organization for Standardization, 2014)]. The soil was placed within a plastic container having internal dimensions of W 283 mm × H 48 mm × L 193 mm, atop which the film samples were arranged (Figure 1B). The samples were then covered by more soil. Several containers of the samples were thus prepared and placed in an incubator (CN-40A, Mitsubishi Electric Engineering), following which the biodegradation process was started. The temperature was maintained at 28°C as aerobic microorganisms are most active in this environment (Pietikäinen et al., 2005).

After a designated biodegradation time (0–24 h), the containers were removed from the incubator, and the film samples were carefully extracted (Figure 1C). To stop the activity of the microorganisms adhering to the surfaces, each sample was sterilized by irradiating with ultraviolet (UV) light on both sides (1.5 min on each side). Subsequently, the extra moisture was removed from the samples by drying in a vacuum oven (ADP300, Yamato Science) at 40°C for 1 h. Thereafter, the surfaces of the samples were cleaned carefully using a sponge moistened with a small amount of distilled water. Finally, the film samples were irradiated again with UV light (1.5 min on each side) and stored in the humidity chamber until the characterization experiments. During these steps, we did our best to minimize the time required to move the samples in and out of the chamber and incubator and clean them.

### Characterization of the Film Samples

#### Tensile Testing and Acquisition of the Mechanical Properties

**FIGURE 2:**
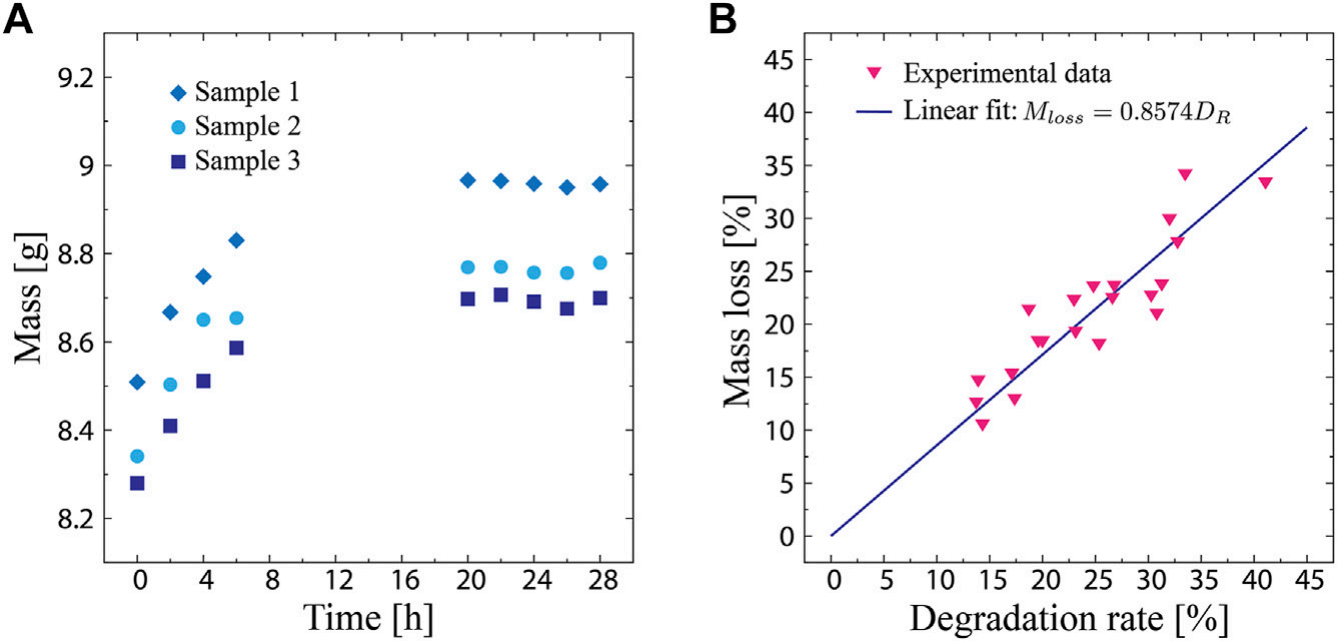
**(A)** Sample mass in the humidity chamber as a function of the elapsed time. **(B)** Sample mass loss as a function of the biodegradation rate, which is defined as the change in moisture content.

Since the mechanical properties of the samples can be changed by the moisture in the air, the samples were placed in a regulated environment in a humidity chamber for at least 24 h at 67% RH, at which time the elongation at breakage of a film made from gelatin and glycerol is maximal (Lim et al., 1999). The film samples were punched out into dumbbell shapes (JIS K6251, ISO 37, Type 1A (ISO 37:2017. International Organization for Standardization, 2017)), as shown in Figure 1D. Three tensile test specimens were obtained from each sample, and the thickness of each test piece was measured using a laser displacement sensor (CDX-L15, OPTEX FA). The median of the measured values from three different locations on the sample were considered. Following the thickness measurement, the test pieces were returned to the humidity chamber and stored in an equilibrium state for more than 24 h, as shown in Figure 2A, which shows the sample mass at this stage. Uniaxial tensile tests were then performed on all the specimens using a universal testing machine (AGS-20NX, Shimadzu), and the stress–strain curves were recorded. This test was performed at a tensile speed of 50 mm/min until the specimen broke. During the tests, the room was maintained at ∼ 60% RH using a humidifier (temperature ∼24°C). Figure 1E displays a specimen undergoing tensile testing. The data acquired from the tensile tests were analyzed to determine the mechanical properties of the film samples. We chose to investigate the elongation at breakage, Young’s modulus, and material constants of the Yeoh hyperelastic material model (Yeoh, 1993). We selected this model because it is known to be suitable for both small and large strains (Ali et al., 2010). In addition, other hyperelastic material models, such as Ogden and Gent, may also be useful because of their accuracy and compatibility with the finite element analysis software (Wex et al., 2015). The Yeoh model takes the form of a strain energy density function given by

$$\begin{array}{c} W=\overset{3}{\underset{i=1}{\sum }}C_{i}{\left(I_{1}-3\right)}^{i} \end{array}.$$ (1)

where $C_{i}$ is a material constants and $I_{1}$ is the strain invariant ( $I_{1}=\lambda_{1}^{2}+\lambda_{2}^{2}+\lambda_{3}^{2}$ ). $\lambda_{1}$ , $\lambda_{2}$ , and $\lambda_{3}$ are the stretch ratios in the length, width, and thickness directions, respectively. Assuming that the material is incompressible ( $\lambda_{1}\lambda_{2}\lambda_{3}=1$ ), the stress along the length direction (tensile direction) is expressed as

$$\begin{array}{c} \sigma_{1}=\lambda_{1}\frac{\partial W}{\partial I_{1}}=2\left(\lambda_{1}^{2}-\frac{1}{\lambda_{1}}\right)\overset{3}{\underset{i=1}{\sum }}iC_{i}{\left(\lambda_{1}^{2}+\frac{2}{\lambda_{1}}-3\right)}^{i-1} \end{array}.$$ (2)

By fitting Eq. 2 to the measured stress–strain curve, the material constants $C_{1}$ , $C_{2}$ , and $C_{3}$ are obtained. The Young’s modulus of the material Y is then obtained using the relationship $Y=6C_{1}$ .

#### Acquisition of the Degradation Rate

The degradation rate of a material depends on its environmental factors, such as the activity of the microorganisms, moisture, and temperature (Linn and Doran, 1984; Pietikäinen et al., 2005), making it difficult to determine the rate of degradation of the samples using only the degradation time. In some studies, the biodegradation of the materials was evaluated based on the weight loss before and after degradation (Martucci and Ruseckaite, 2009a; González et al., 2011). However, owing to strong adhesion, some of the soil often remained on the samples prepared in this study, preventing accurate measurement of the mass change. In addition, the same issue has been pointed out in the literature for gelatin (Martucci and Ruseckaite, 2009b). A previous study on wood pulp fibers showed that the water retention value decreased as biodegradation increased (Yamagishi and Oye, 1981). We believe that the same mechanism occurred in our gelatin and glycerol mixture. Depending on the amount of degradation, gelatin polymer chains break and lose water that can be retained. Based on this assumption, the following equation was used to calculate the degradation rates of the samples $D_{R}$ in this study.

$$\begin{array}{c} D_{R}\;\left[\%\right]=\;-\frac{M_{C}-M_{C0}}{M_{C0}}\times 100 \end{array},$$ (3)

where, $M_{C0}$ is the average moisture content of the samples before degradation, and $M_{C}$ is the average moisture content of the samples after the degradation. To measure the moisture content, a single sample of each test membrane was reserved after punching out the tensile test specimens. The samples for estimating the degradation rate were equilibrated at 67% RH for more than 24 h together with the tensile test specimens. Immediately after the tensile tests, the masses of the samples $M_{0}$ were measured. Then, the corresponding samples were dried at 110°C for 24 h in an oven. Subsequently, the masses of the dried samples $M_{d}$ were measured. Finally, the moisture content of the sample $M_{C}$ was determined by the following equation.

$$\begin{array}{c} M_{C}\;\left[\%\right]=\frac{M_{0}-M_{d}}{M_{0}}\times 100 \end{array}.$$ (4)

#### Validity of the Degradation Rate

To confirm the validity of the degradation rate, which is defined by Eq. 3, a preliminary control experiment was conducted. Bromelain, an enzyme extracted from pineapples, catalyzes the hydrolysis of gelatin (Gautam et al., 2010; Amid et al., 2011; Choonpicharn et al., 2015), resulting in mass loss, which is a common metric for determining the degree of biodegradation (Dalev et al., 2000; Martucci and Ruseckaite, 2009b). The mass loss is expressed as

$$\begin{array}{c} M_{loss}\;\left[\%\right]=-\frac{M^{\prime }-M_{0}^{\prime }}{M_{0}^{\prime }}\times 100 \end{array},$$ (5)

where $M_{0}^{'}$ and $M^{'}$ are the sample masses before and after degradation, respectively.

Membrane samples composed of the gelatin and glycerol mixture were cut into squares of 20 mm × 20 mm (thickness: ∼1 mm) and dried in a vacuum oven at 40°C for 24 h. The mass of the samples corresponding to $M_{0}^{'}$ was then measured. Afterward, the samples were equilibrated in the humidity chamber for 24 h at 67% RH. Subsequently, their mass at equilibrium state ( $M_{e1}$ ) was measured again to obtain the moisture content before degradation ( $M_{C0}$ ), which was calculated as $M_{C0}=\left(M_{e1}-M_{0}^{'}\right)/M_{0}^{'}$ . Thereafter, the samples were immersed in a bromelain solution at room temperature (∼24°C) for 5, 10, 15, 20, 25, 30, and 60 min to catalyze degradation. Three samples were used for each time period. The bromelain solution was prepared by extracting juice from commercial pineapples using a manual fruit press and filtering it through a paper filter. After each degradation time, the corresponding sample was rinsed with distilled water, sterilized with UV irradiation for 3 min, and dried in a vacuum oven at 40°C for 24 h. Subsequently, the mass of the sample corresponding to $M^{'}$ was measured, and the sample was equilibrated in the humidity chamber for 24 h at 67% RH. The sample mass at equilibrium state ( $M_{e2}$ ) was measured, and the moisture content after the degradation ( $M_{C}$ ) was calculated as $M_{C}=\left(M_{e2}-M^{'}\right)/M^{'}$ . Finally, the mass loss and corresponding biodegradation rate were obtained using Eqs 5, 3, respectively.

Figure 2B shows the mass loss of the samples plotted as a function of the biodegradation rate. The experimental data reveal a linear correlation between the mass loss and biodegradation rate, where the mass loss increases as the biodegradation rate increases. This indicates that a change in the moisture content can represent the degree of biodegradation, validating Eq. 3. By performing a linear regression on the data, the expression of their relationship was obtained as follows:

$$\begin{array}{c} M_{loss}\;=0.8574D_{R} \end{array}.$$ (6)

### Finite Element Analysis and Fabrication of the Soft Actuator

A finite element analysis software (ABAQUS, Dassault Systems) was used as the simulation environment to predict the behavior of a pneumatic, biodegradable soft actuator. The geometric model of the pneumatic soft actuator was created using a CAD software (SOLIDWORKS, Dassault Systems). In the simulations, the material properties of the model were set as the constants of the Yeoh model ( $C_{1}$ , $C_{2}$ , and $C_{3}$ ), as described in *Characterization of the Film Samples*. These material constants were implemented as parameters in the function of the degradation rate to simulate the behavior of the actuator for input pressure under the biodegradation process.

**FIGURE 3:**
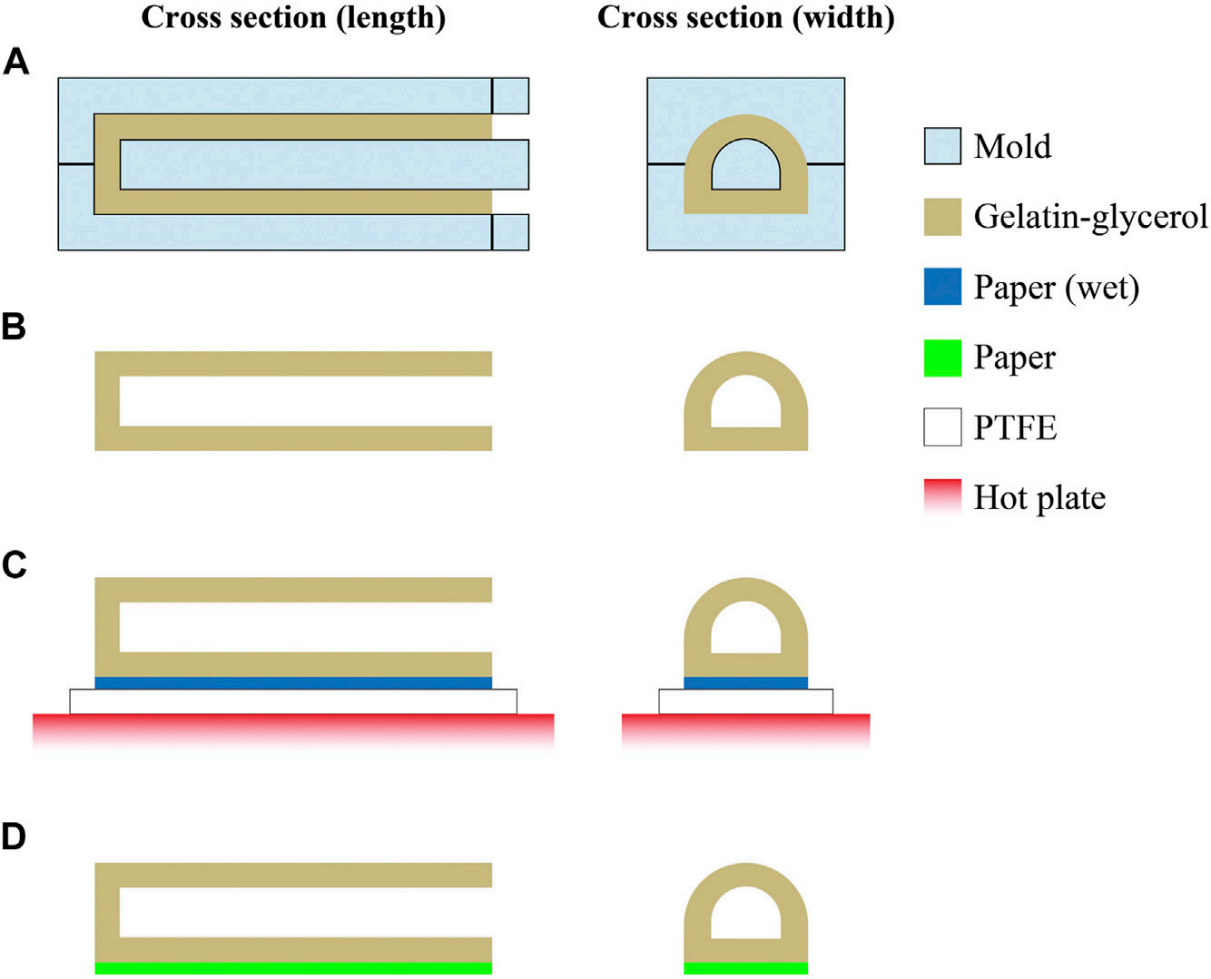
Fabrication of the physical soft actuator. **(A)** Gelatin–glycerol mixture is poured into a mold. **(B)** After curing, the mold is detached from the actuator structure. **(C)** A wet paper was placed at the bottom of the actuator structure and heated using a hot plate for bonding; polytetrafluoroethylene (PTFE) was placed between the paper and hot plate to avoid adhesion. **(D)** After heating the sample, the actuator with the paper as a strain-limiting layer is fabricated.

**FIGURE 4:**
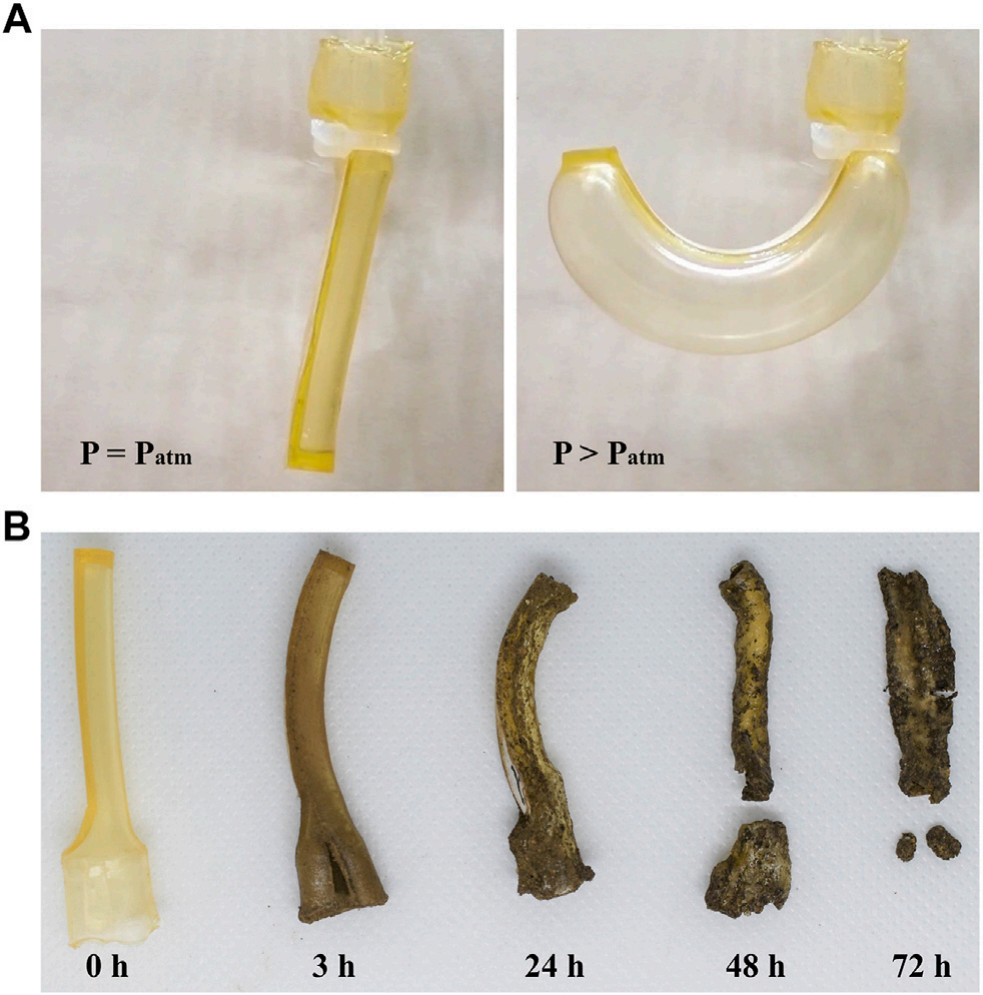
Biodegradable actuator fabricated in this study. **(A)** Actuator under non-pressurized and pressurized states. **(B)** Actuator under biodegradation process.

Figure 3 details the fabrication process of the physical actuator that was produced by pouring the mixture described in *Preparation of Film Samples* into a 3D printed mold. The actuator mold was designed using CAD software and fabricated with a 3D printer (Form 3, Formlabs). The actuator was then cured at 40°C for 2 h in an oven. Thereafter, a wet paper was bonded to the bottom of the actuator using a hot plate, followed by drying in the vacuum oven at 40°C for 12 h. The paper acts as a strain-limiting layer that ensures bending of the actuator structure when pressurized. Subsequently, the device was maintained in a humidity chamber (WET-297-AHU, Tolihan) whose moisture content was equilibrated at 67% RH. Figure 4 shows the fabricated actuator.

### Scaling of the Model

In the fabrication process described in the previous section, the mold is designed such that the actuator has a total length of 50 mm and width of 10 mm. However, after the equilibration process in the humidity chamber, the change in the water content of the actuator causes a reduction of its volume. This means that the size of actuator is different from that of the mold. To account for this volume reduction, the actuator model was scaled in the simulation environment. The scaling factor, α , was defined from the volume ratio before and after curing; α was found to be 0.62 ± 0.07 (*N* = 4) from the measurements and calculations described below. We consider films formed using a mixture of gelatin and glycerol poured into molds and cured. We define the densities of the gelatin, glycerol, and water as $\rho_{Gel}$ , $\rho_{Gly}$ , and $\rho_{W}$ , and their volume after curing as $V_{Gel}$ , $V_{Gly}$ , and $V_{1_{W}}$ , respectively. The mass of the film $m_{1}$ and its volume $V_{1}$ are expressed as

$$\begin{array}{c} m_{1}=\rho_{Gel}V_{Gel}+\;\rho_{Gly}V_{Gly}+\rho_{W}V_{1_{W}} \end{array},$$ (7)

$$\begin{array}{c} V_{1}=V_{Gel}+V_{Gly}+V_{1_{W}} \end{array}.$$ (8)

The films were then equilibrated in the humidity chamber during the conditioning process. By assuming that only the volume of water was changed, the volume of water after equilibration $V_{2_{W}}$ and its mass $m_{2}$ are expressed by the following relationship.

$$\begin{array}{c} m_{2}=\rho_{Gel}V_{Gel}+\;\rho_{Gly}V_{Gly}+\rho_{W}V_{2_{W}} \end{array},$$ (9)

$$\begin{array}{c} V_{2}=V_{Gel}+V_{Gly}+V_{2_{W}} \end{array}.$$ (10)

where $V_{2}$ is the volume of the film after conditioning. In this state, the volume reduction of the film β is expressed as

$$\begin{array}{c} \beta =\frac{V_{2}-V_{1}}{V_{1}}=\frac{V_{2_{W}}-V_{1_{W}}}{V_{1}}=\frac{m_{2}-m_{1}}{\rho_{W}}\times \frac{1}{V_{1}} \end{array}.$$ (11)

Let the masses of gelatin, glycerol, and water before mixing be $M_{Gel}$ , $M_{Gly}$ , and $M_{0W}$ , respectively, and the total mass of the solution after mixing be $M_{0}$ . Assuming that gelatin and glycerol do not evaporate, the mass ratio is expressed as

$$\begin{array}{c} M_{0_{Gel}}\;:M_{0_{Gly}}\;:M_{0_{W}}=M_{Gel}\;:M_{Gly}\;:M_{0}-M_{Gel}-M_{Gly} \end{array}.$$ (12)

The mass ratio of the materials in the mixture contained in the mold is

$$\begin{array}{c} m_{0_{Gel}}\;:m_{0_{Gly}}\;:m_{0_{w}}=\frac{M_{Gel}}{M_{0}}m_{0}\;:\frac{M_{Gly}}{M_{0}}m_{0}\;:\frac{M_{0}-M_{Gel}-M_{Gly}}{M_{0}}m_{0} \end{array},.$$ (13)

where $m_{0}$ is the mass of the mixture in the mold, and $m_{0_{Gel}}$ , $m_{0_{Gly}}$ , and $m_{0_{w}}$ are the masses of gelatin, glycerol, and water in the mold, respectively. After curing, the mass of water is reduced by $m_{0}-m_{1}$ . The mass ratio of the materials in the mixture after curing is

$$\begin{array}{c} m_{1_{Gel}}\;:m_{1_{Gly}}\;:m_{1_{W}}=\frac{M_{Gel}}{M_{0}}m_{0}\;:\frac{M_{Gly}}{M_{0}}m_{0}\;:\frac{M_{0}-M_{Gel}-M_{Gly}}{M_{0}}m_{0}-\left(m_{0}-m_{1}\right) \end{array},$$ (14)

where $m_{1_{Gel}}$ , $m_{1_{Gly}}$ , and $m_{1_{w}}$ are the masses of gelatin, glycerol, and water after curing. The density of the film $\rho_{1}$ in this state is estimated by the following equation:

$$\begin{array}{l} \rho_{1}=\frac{\frac{M_{Gel}}{M_{0}}m_{0}\rho_{Gel}+\frac{M_{Gly}}{M_{0}}m_{0}\rho_{Gly}+\left\{\frac{M_{0}-M_{Gel}-M_{Gly}}{M_{0}}m_{0}-\left(m_{0}-m_{1}\right)\right\}\rho_{W}}{m_{1}}\\ \;\;\;\;\;\;\;=\frac{m_{0}}{M_{0}m_{1}}\left\{M_{Gel}\rho_{Gel}+M_{Gly}\rho_{Gly}+\left(M_{0}\frac{m_{1}}{m_{0}}-M_{Gel}-M_{Gly}\right)\rho_{W}\right\} \end{array}.$$ (15)

Since $V_{1}\;=\;m_{1}/\rho_{1}$ , the volume reduction of the film β is given as

$$\begin{array}{c} \beta =\frac{m_{2}-m_{1}}{\rho_{W}}\times \frac{m_{0}}{M_{0}m_{1}^{2}}\left\{M_{Gel}\rho_{Gel}+M_{Gly}\rho_{Gly}+\left(M_{0}\frac{m_{1}}{m_{0}}-M_{Gel}-M_{Gly}\right)\rho_{W}\right\} \end{array}.$$ (16)

Finally, the scaling factor α is derived as

$$\begin{array}{c} \alpha =\frac{V}{V_{0}}=\frac{V_{0}}{V_{0}}+\frac{V-V_{0}}{V_{0}}=1+\beta \end{array}$$ (17)

### Validation of the Simulation Model

The physical actuator was affixed to a setup consisting of a pump, microcontroller, solenoid valve, and pressure sensor, to which up to 240 kPa of air pressure was applied. During the experiment, the input pressure was applied in increments of 20 kPa. Figure 4A shows the actuator in the non-pressurized and pressurized states. As the pressure increases, the amount of bending deformation increases. Figure 4B displays the ability of the actuator to return to the soil by decay. Given a sufficient amount of biodegradation time, the device disintegrates and blends into the soil by microbial activity.

**FIGURE 5:**
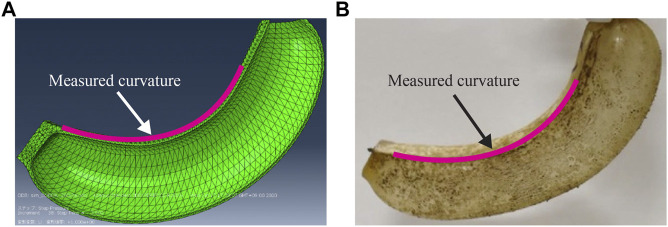
Pressurized bending deformation of **(A)** simulated and **(B)** physical actuators (applied pressure: 240 kPa, biodegradation rate: 5%).

To validate the simulation model through characterization of the physical actuator, the pressurized deformation under the degradation process was captured using a camera (D7500, Nikon). From the captured image, the actuation curvature was measured, as indicated in Figure 5. For this purpose, an image processing software (Kappa plugin (Mary and Brouhard, 2019) for Fiji package which is distribution of ImageJ2) was used. The curvature of the simulated actuator was obtained in a similar manner. In this experiment, three samples were prepared with target degradation rates of 0, 5, 10, 15, 20, and 25%. The conditions for the biodegradation process of the actuator and acquisition of degradation rate are identical to those explained in *Biodegradation of Film Samples* and *Characterization of the Film Samples*. During this experiment, the room was maintained at ∼ 60% RH using a humidifier (temperature ∼24°C).

## Results and Discussion

### Characterization of the Film Samples

**FIGURE 6:**
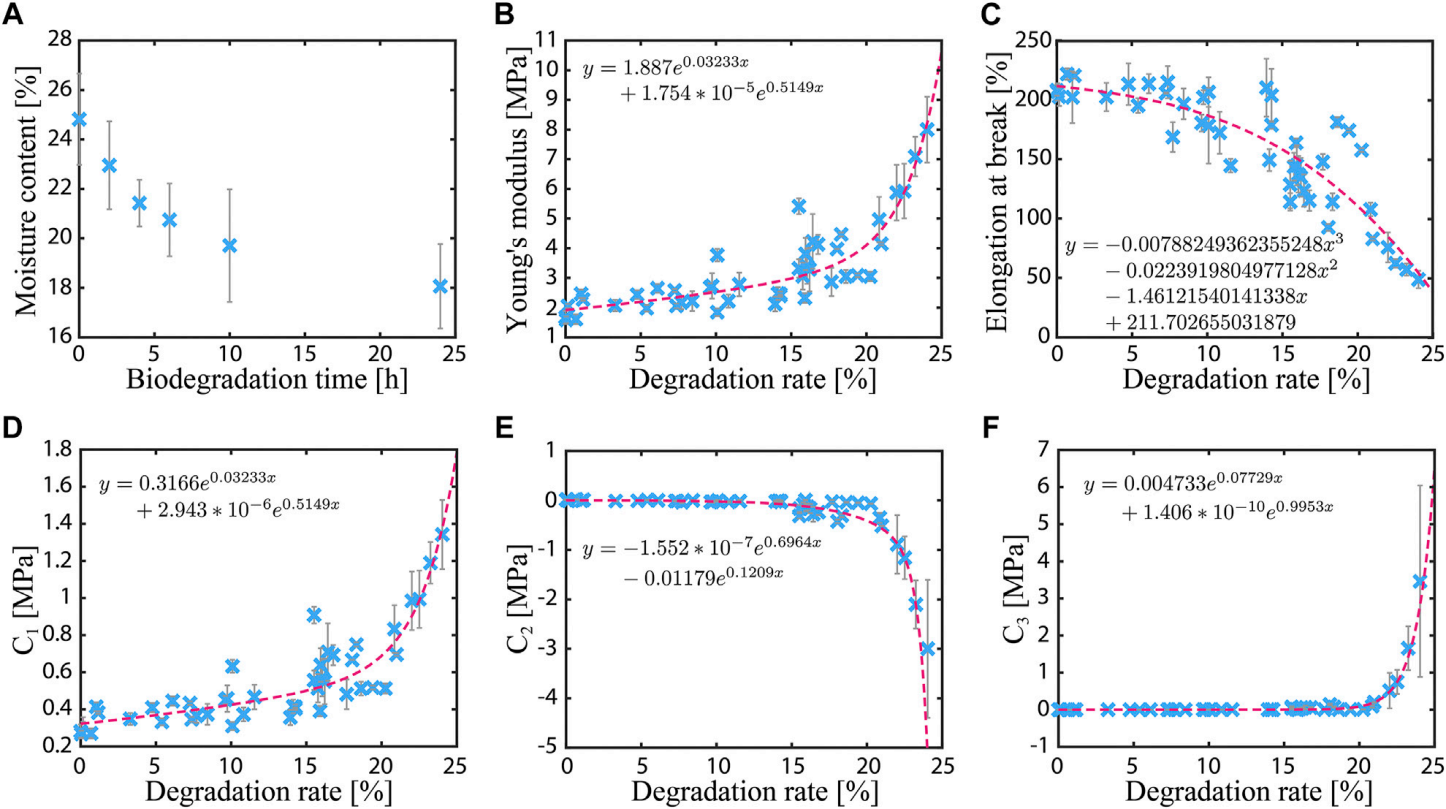
Characterization results of the gelatin–glycerol films. **(A)** Moisture content of the film sample as a function of the degradation time. **(B, C)** Young’s modulus and elongation at break as functions of the degradation rate. **(D–F)** Material constants of the Yeoh hyperelastic material model as functions of the degradation rate.

The relationship between degradation time and moisture content of the film samples is shown in Figure 6A. The data are the average values of the samples at each degradation time (*N* = 9). The moisture content of the samples decrease with increasing degradation time because of the separation of the gelatin molecule chains forming the network structure, thereby resulting in reduction of space for storing water. The slope becomes more gradual as the degradation time increases, which may have resulted from inhibition of microbial activity with increasing material degradation. The results also suggest that the change in the water content represents the biodegradation rate of the material.

A nonlinear trend is observed in the mechanical properties. Figures 6B,C shows the measured Young’s modulus and elongation at break as functions of the degradation rate. The approximate curve (dashed line) in the graph is obtained by the least-squares method. The data are the average values of three samples. During the tests, a similar failure mode was observed in the samples regardless of the biodegradation rate. Every sample fractured at the narrow part of the dumbbell shape. In these broken samples, the fracture cross-section is smooth up to 20% of the biodegradation rate. At 25%, the cross-section is slightly jaggy, like ragged textiles.

The Young’s modulus increases with increasing degradation rate. At 25% degradation, the modulus is ∼5.5 times larger than the value at the initial state (1.73 ± 0.15 MPa). On the other hand, the elongation at break decreases with increasing degradation rate. At 25% degradation, the elongation is ∼4 times smaller than the initial value (209.8 ± 6.3%). In addition, the material constants of the Yeoh model, i.e., $C_{1}$ , $C_{2}$ , and $C_{3}$ , obtained by fitting the measured stress–strain curve show similar quadratic trends (Figures 6D–F). These changes in the mechanical properties and coefficients are owed to the gelatin molecule chains being cut by the action of microorganisms during degradation, thereby causing collapse of the polymer network and reduction of the flexibility. The results show clear relationships between the mechanical properties and degradation progression of the soft biodegradable material. In addition, the negative value of $C_{2}$ might make the model unstable; however, as we will describe in a later section, no instability was observed. This is because the value of $C_{2}$ is considerably small at lower degradation rates (i.e., large actuation strain), whereas it increases at higher degradation rates (i.e., small actuation strain). Depending on the type of material and its actuation behavior, it might be problematic to have a negative $C_{2}$ . In that case, fitting can be done by setting a positive boundary condition of $C_{2}$ .

Even though no visible weak spots were observed in the samples during the experiments, we assume that the damage caused by biodegradation is not perfectly homogeneous on a microscopic scale. This is because the local microbial activity was slightly different across the sample area. However, since the difference is subtle, we assume that the biodegradation is macroscopically homogeneous.

### Validation of the Simulation Model

**FIGURE 7:**
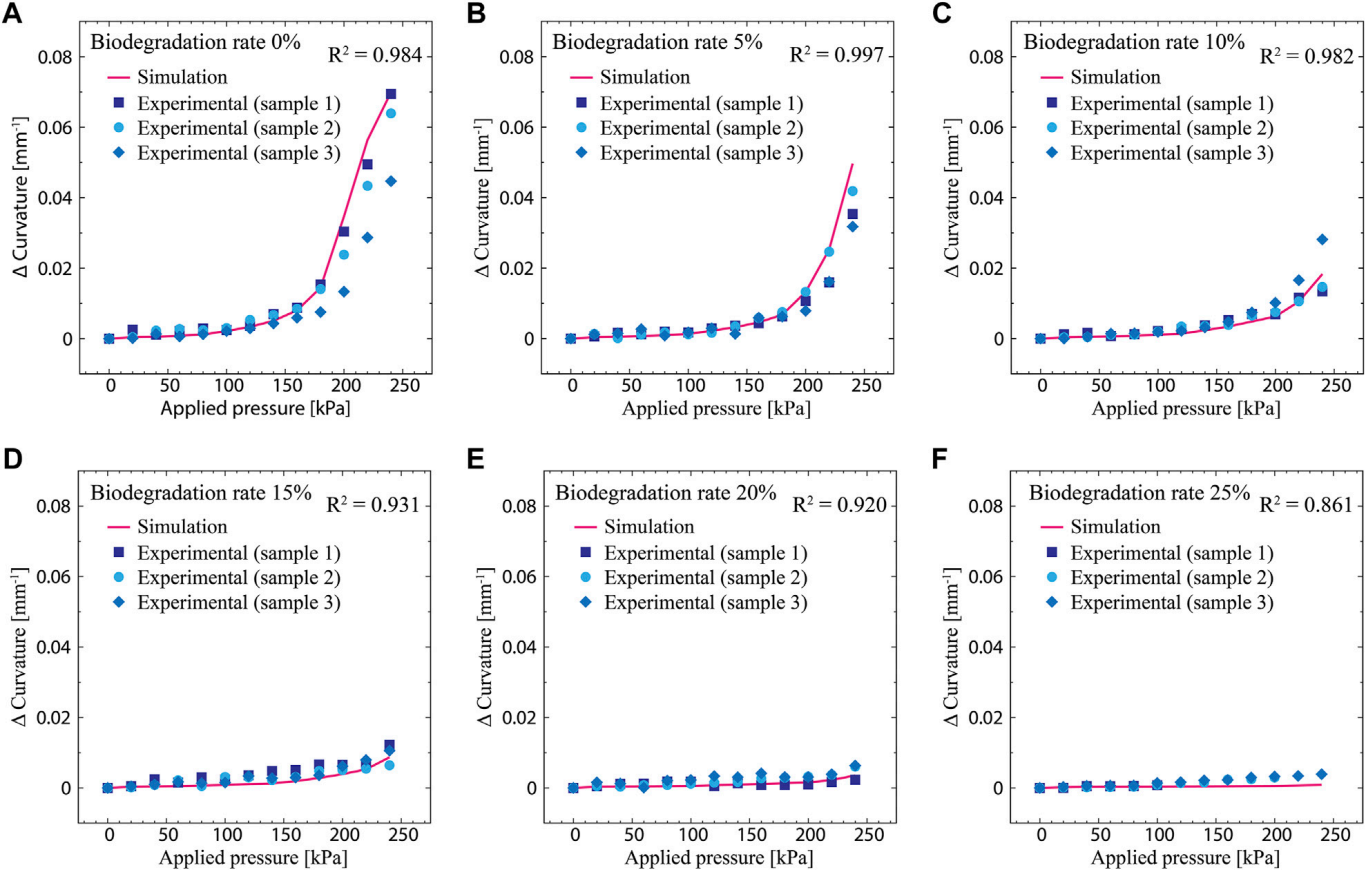
Actuation curvatures as functions of the applied pressure measured from the simulated model and physical actuator; the curvature is a relative value from the initial state. **(A)** Degradation rate of 0%. **(B)** Degradation rate of 5%. **(C)** Degradation rate of 10%. **(D)** Degradation rate of 15%. **(E)** Degradation rate of 20%. **(F)** Degradation rate of 25%.

Figure 7 depicts the simulated actuation curvature as a function of the applied pressure for different degradation rates: 0, 5, 10, 15, 20, and 25%. Note that with the exception of the 0% case, the degradation rates of the physical actuator are slightly different from the simulated values. This is attributed to the fact that it is almost impossible to exactly degrade the physical actuator to a desired rate. In Figure 7, the curvature is plotted as a relative value from the initial state. Overall, the actuation curvature increases with increasing applied pressure. As the degradation rate increases, the actuation curvature at a given applied pressure decreases. The reduction of the actuation curvature is attributed to stiffening of the degraded material, as shown in Figure 6B. The change in the elongation at break also influences the actuation performance. As can be seen in Figure 7F (degradation rate of 25%), two of the three samples tested experienced ruptures before reaching the maximum pressure. This is due to the shortened elongation at break that limits the actuated deformation, which renders the actuator fragile.

The simulation model is observed to exhibit the same trend as that observed in the physical actuator. As the applied pressure increases, the actuation curvature also increases; as the degradation rate increases, the actuation curvature decreases at a given applied pressure. Comparisons of the simulated and experimental results show good agreement. Overall, the simulated model predicts the behavior of the physical actuator well under biodegradation. The *R* ^2^ values between the models and measured data are calculated to be 0.984, 0.997, 0.982, 0.931, 0.920, and 0.861 for degradation rates of 0, 5, 10, 15, 20, and 25%, respectively. The error between the simulated and experimental results are attributed to slight differences in the degradation rates between the physical actuator and model. Additionally, the degradation of the structure of the physical actuator may be inhomogeneous, which could lead to discrepancies in the simulation model. Even though the room humidity was maintained constant with a humidifier (60% RH), there may be subtle changes in humidity, which may change the water content of the actuator and therefore its compliance. This may be the reason why errors are observed, especially in the case of 0% degradation rate.

In the results noted above, the experimental data validate the adequacy of the simulation model and confirm that the actuation behaviors of biodegradable soft actuators change as a function of their degradation rates, whose characteristics can be predicted. The results also suggest that the desired outputs of the actuators can be achieved under biodegradation and that the proposed approach and implementation of the experimental dataset in the simulated environment are effective for designing and controlling biodegradable soft actuators.

## Conclusion

We present a framework to design biodegradable soft actuators and predict their actuation behaviors under degradation. We also characterize a mixture of gelatin and glycerol as a biodegradable material and build the simulation environment by implementing experimentally determined material constants. The materials used in this study show that as the rate of biodegradation increases, the Young’s modulus increases and elongation at break decreases. We clarify that the changes in these characteristics influence the behaviors of the physical actuators; the higher the biodegradation rates, the more reduced are the actuation curvatures. We also confirm that the behaviors of degraded actuators can be predicted adequately with the developed simulation model, which illustrates the applicability of the framework to the design and control of biodegradable soft actuators and robots.

Further evaluation and implementation of mechanical and material properties, such as failure limits in different loading conditions (e.g., biaxial and multiaxial loadings), viscoelasticity, and creep, will be conducted in the future with respect to the investigation presented here. Given the normal stress hypothesis (Rosendahl et al., 2019), the experimental data in the current study provide sufficient information to understand failure limits in various loading conditions. Further characterization will improve its accuracy and provide additional insight into the behavior of the biodegradable material. This type of experiment is particularly important when the material is filled with additives to modify the properties, such as improving environmental tolerance, which is expected to occur when biodegradable robots are used in practical situations.

In future experiments, various combinations of environmental factors, such as temperature, humidity, and soil composition, will be considered in the characterizations and simulations to simulate a more natural environment. This will result in the creation of a data set that can be used to match predictions to a specific working environment. Regarding the characterization, the degradation rate should be determined not only from changes in the moisture content but also from the mass losses of materials (Dalev et al., 2001; Abd El-Rehim et al., 2004; Martucci and Ruseckaite, 2009a; González et al., 2011), change in the molecular mass (Albertsson et al., 1998; Jakubowicz, 2003), or increase in CO_2_ due to respiration by microorganisms (Linn and Doran, 1984; Pietikäinen et al., 2005). Since the characterization and simulation processes established in this study are versatile, it is expected that our framework can be used to test various soft robotic biodegradable materials. In these tests, an adequate method for determining the degradation rate should be selected for each target material. Additionally, other biodegradation methods, such as UV irradiation and enzymes, can be adopted. It is also important to investigate the effectiveness of the proposed strategy for soft actuators and robots of various types and shapes. Integrating other biodegradable mechatronic elements, such as sensors, batteries (Irimia-Vladu et al., 2012; Irimia-Vladu, 2014; Tan et al., 2016; Zhu et al., 2016), and a pneumatic power source (Okui et al., 2017), in future device developments will significantly increase functionality and enable significant research results. Upon examination of the works mentioned above, our strategy is expected to become a powerful design and control method for soft actuators and robots based on diverse biodegradable materials as well as contribute to the formulation of green robotics.

## Data Availability

The raw data supporting the conclusions of this article will be made available by the authors, without undue reservation.
